# Inflammation-induced hypoparathyroidism triggered by combination immune checkpoint blockade for melanoma

**DOI:** 10.1186/s40425-019-0528-x

**Published:** 2019-02-21

**Authors:** Beckey Trinh, Guacimara Ortega Sanchez, Petra Herzig, Heinz Läubli

**Affiliations:** 1 Clinic of Endocrinology, Diabetes and Metabolism, Basel, Switzerland; 2 Department of Internal Medicine, Division of Medical Oncology, Basel, Switzerland; 3 Department of Biomedicine, Cancer Immunology Laboratory, Basel, Switzerland; 4 Department of Clinical Research, University Hospital Basel, Petersgraben 4, 4031 Basel, Switzerland

**Keywords:** Parathyroid hormone, Immune-related adverse event, Hypocalcemia

## Abstract

**Background:**

Treatment with a combination of PD-1 and CTLA-4 targeted checkpoint inhibition has improved outcome of melanoma patients and led to durable remissions but is also associated with significant toxicities. Endocrinopathies such as thyroiditis and hypophysitis are often seen, but other, rarer disturbances have also been described. Endocrinopathies affecting the parathyroid gland are rarely reported and no clear pathomechanism has been proposed.

**Case presentation:**

Here, we report a case of severe hypocalcemia due to an antibody-mediated hypoparathyroidism as an immune-related adverse event (irAE) in a patient who was treated with the anti-PD-1 antibody nivolumab and anti-CTLA-4 antibody ipilimumab. Hypocalcemia was rapidly corrected by substitution, but the endogenous serum parathyroid hormone (PTH) remained low. The patient demonstrated a rapid and profound tumor response to the combination immune checkpoint blockade, but developed a severe colitis that required high-dose intravenous corticosteroid and anti-TNFα therapy. During this strong immunosuppression the PTH level normalized and the calcium levels were stable without substitution. However, during tapering of immunosuppressants, the PTH and calcium levels decreased again to a level requiring calcium substitution.

**Conclusion:**

Our report demonstrates a rare endocrinopathy as a complication of combined PD-1 and CTLA-4 blockade. In addition, it provides evidence from the course of the disease that inflammation within the parathyroid gland is involved in the mechanism.

## Background

Nivolumab, a programmed cell death protein 1 (PD-1) antibody, and ipilimumab, a cytotoxic T-lymphocyte antigen 4 (CTLA-4) antibody, are immune checkpoint inhibitors (ICI) which have emerged as first- and second-line therapy of stage IV melanoma [1, 2]. However, they can induce immune-related adverse events (irAEs) involving a range of organs similar to auto-immune diseases [1, 3, 4]. The endocrine system is often affected [1, 3, 4], with disturbances of the thyroid and the pituitary gland being the most common endocrinopathies [1]. Inflammation of the thyroid gland can often lead to an initial hyperthyroidism and subsequent hypothyroidism. Inflammation of the pituitary gland can lead to insufficiencies of the thyroid, adrenal and gonadal axis. Also, rarer endocrinopathies such as adrenalitis have been described, but the course of such inflammations and also the pathomechanisms are less well-known.

## Case report

**Fig. 1:**
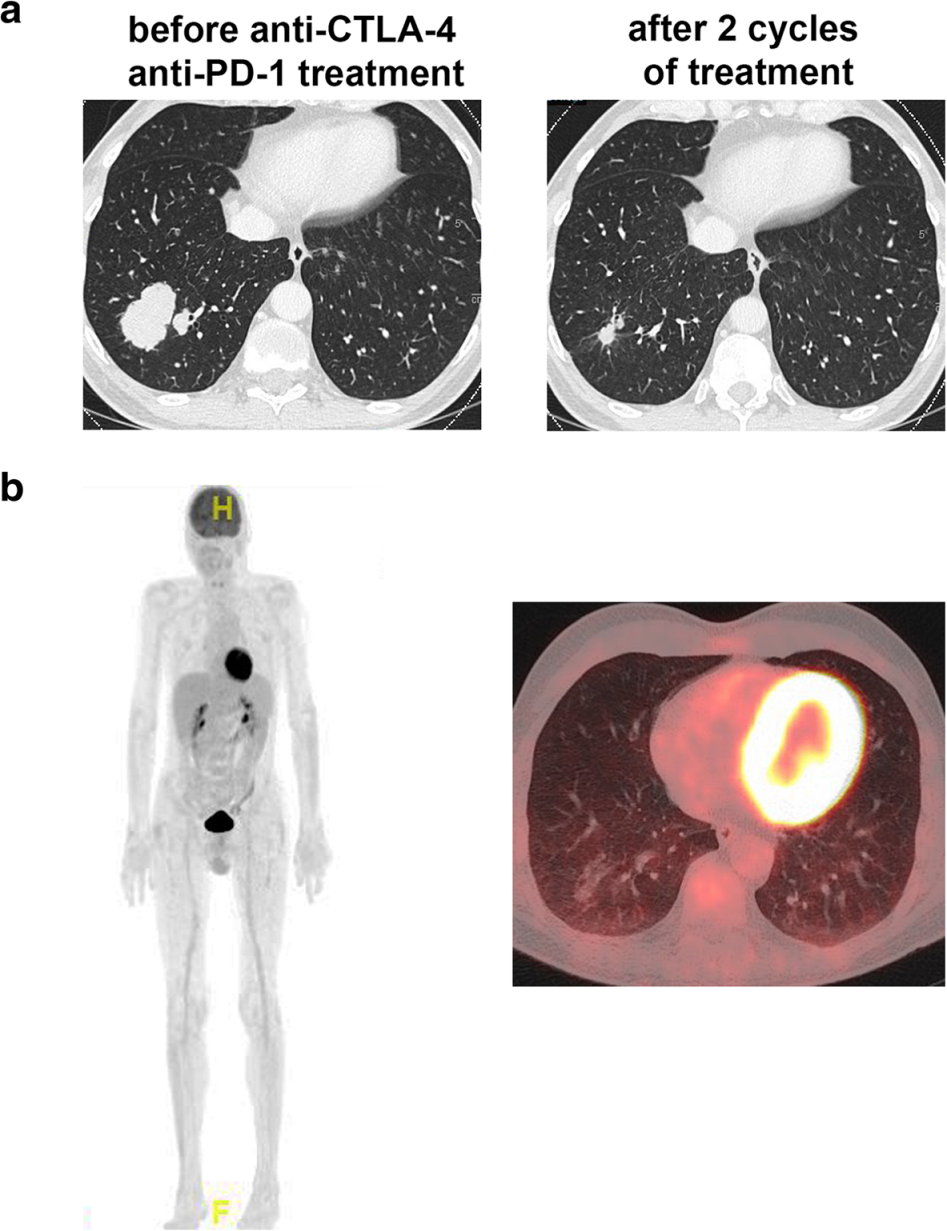
Clinical response to nivolumab and ipilimumab. **a**, Lung lesion before the treatment with anti-CTLA-4 and anti-PD-1 antibodies and after 8 weeks. There was a marked regression with 8 weeks of the treatement. **b**, After 16 weeks of treatment, a complete metabolic response was observed in the ^18^FDG-PET/CT scan

Here, we report on a 53-year old patient with stage IV melanoma who was admitted to the hospital after he presented with generalized paresthesia, stiffness in both hands, a feeling of obstruction in his throat and mild dizziness. He had metastases in the liver, spleen, lung and bones but not in the brain (Fig. 1a). Treatment of his melanoma with nivolumab 1 mg/kg and ipilimumab 3 mg/kg was started 4 weeks earlier and two doses had been given before the time point of presentation. He reported a significant worsening of symptoms over the past few days. His medical history included arterial hypertension treated with valsartan and bisoprolol and intermittent stomach complaints treated with pantoprazole. He had no known auto-immune disorders before the treatment with nivolumab and ipilimumab was started. Serologies for hepatitis B and C virus were negative before the start and serum levels of TSH and free T4 were within the normal limits. At presentation, his vital signs showed no abnormalities. Physical examination was unremarkable with negative Chovestek’s and Trousseau’s signs. ECG showed a prolonged corrected QT interval (493 ms). Laboratory testing revealed hypocalcemia (calcium 1,35 mmol/l [normal range 2.10–2.65], albumin 38 g/l [normal range 35–52], ionized calcium 0,7 mmol/l [normal range 1,15-1,3]), marginal hypomagnesemia (0,69 mmol/l [normal range 0,70-1,00]) and hyperphosphatemia (1,75 mmol/l [normal range 0,8-1,5]. Intact parathyroid hormone (iPTH) level was inadequately low (7,2 pg/ml [normal range 15–65]), 25-hydroxy vitamin D_3_ level was just above normal range (121 nmol/l [normal range 13.2–118]), venous blood gas showed normal pH of 7.418. There was no clinical sign of other auto-immune endocrinopathies, thyroid hormone and cortisol levels were normal.

The patient was diagnosed with acute symptomatic hypocalcemia most likely due to immune-mediated hypoparathyroidism and hospitalized for further treatment. He was given a total of 4 g calcium gluconate in divided doses and 3 g of magnesium sulfate (corresponding 12,15 mmol) intravenously. Oral calcitriol and calcium carbonate was started in parallel. Intact parathyroid hormone remained low even after normalization of magnesium. Symptoms subsided after elevation of calcium levels and the patient was discharged on oral treatment. However, two weeks later, he was readmitted with symptoms of hypocalcemia which was triggered by acute vomiting and severe diarrhea (7–8 stools per day, grade 3). No stool pathogens were identified. Colon biopsies revealed regenerative changes of crypt epithelia and focal surface epithelial defects suggestive of an immune-mediated colitis under PD-1 and CTLA-4 blockade. Thus, the patient was treated with prednisone at 2 mg/kg intravenously and upon persistence of symptoms switched to treatment with infliximab (5 mg/kg), which led to a rapid reduction of stool frequency and eventually resolution of the diarrhea. The steroid therapy was tapered slowly over the course of 4 weeks. The infliximab treatment was repeated once after 2 weeks. No further episode of diarrhea occurred. We interpreted the colitis most likely as a consequence of CTLA-4 blockade and therefore decided to continue the PD-1 blocking antibody nivolumab alone. The hypocalcemia was corrected by substitution therapy.

**Fig. 2:**
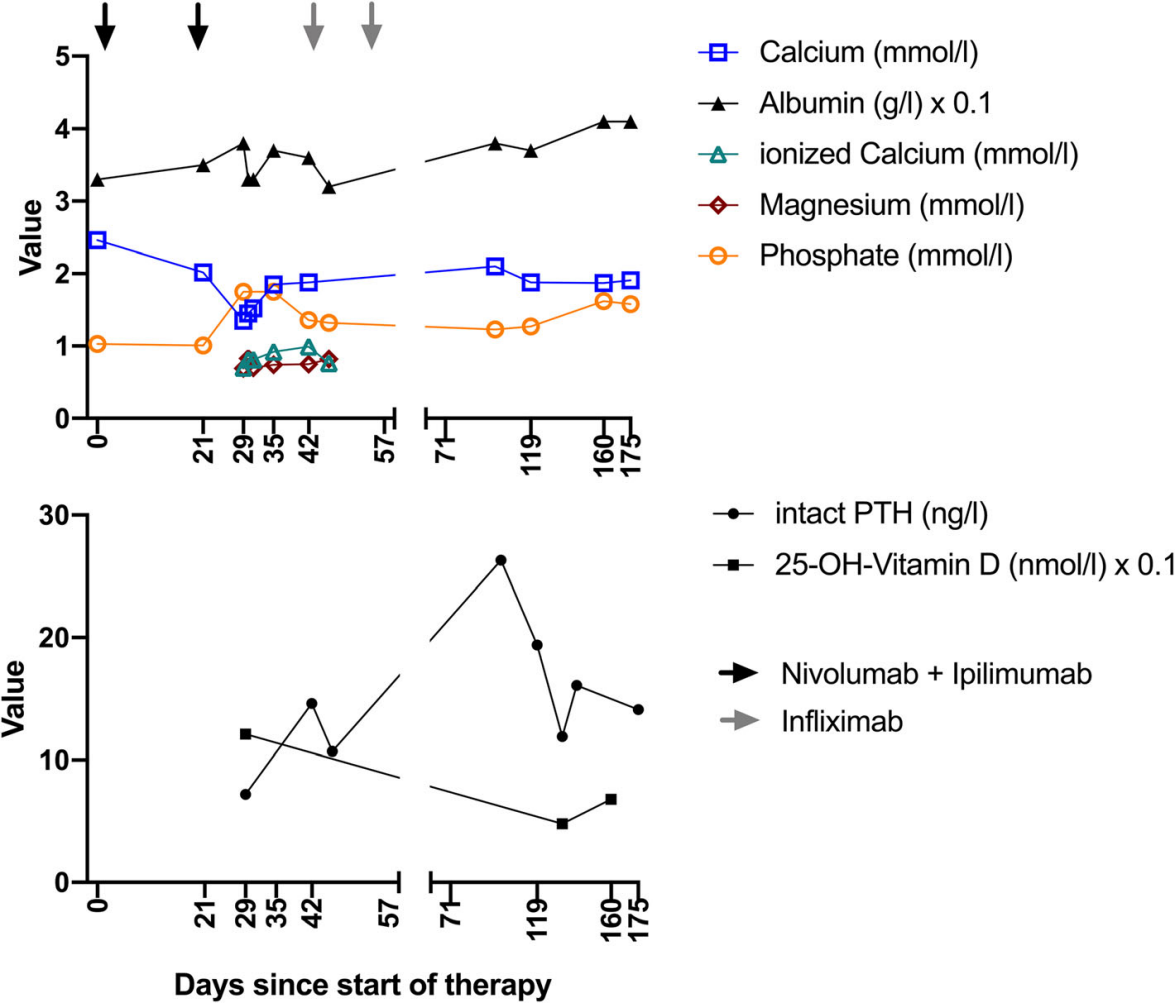
Blood levels of calcium, magnesium, phosphate, intact PTH and 25-OH-vitamin D over the time course of the therapy

^18^F-FDG PET/CT at 5 months after initiation of checkpoint inhibitor therapy showed a complete metabolic remission and an almost complete regression of pulmonary, lymphogenic, splenic and hepatic lesions (Fig. 1b). No enlargement or hypermetabolism was detectable in the typical location of the parathyroid glands. Interestingly, the PTH was increasing during intensified immune suppression with infliximab, but the levels were sinking again during tapering of steroids (Fig. 2). The patient had to be constantly substituted with calcium and in addition, was treated with calcitriol. In order to investigate the reason for the hypoparathyroidism, we analyzed antibodies against the calcium sensing receptor (CaSR). We found detectable levels of auto-antibodies against CaSR in this patient. However, when we analyzed control patients that had also undergone immunotherapy with nivolumab and ipilimumab for melanoma, similar levels of auto-antibodies against CaSR were found, suggesting that the detected antibody concentrations were probably not pathogenic and the test for such low concentrations is most likely unspecific. During the course of the treatment, the patient developed 8 months after start of the immunotherapy an inflammatory oligoarthritis with affection of the right knee and the right ankle. The oligoarthritis was well manageable with local steroid injections. Since a complete response was achieved, we decided at this point to stop the PD-1 blockade. After stopping nivolumab, the calcium substitution could be reduced.

## Discussion

Immune checkpoint inhibitors provide a promising treatment of various cancer entities by enhancement of a patient’s antitumor immunity but are associated with inflammatory side effects. The incidence of any grade irAEs in patients on therapy with ICI is reported to range from 15 to 90% [1, 3, 4]. Immune-mediated endocrinopathies are among the most common irAEs. The most frequently reported immune-mediated endocrinopathies are hypophysitis and thyroid dysfunction while type 1 diabetes mellitus and adrenal insufficiency are less commonly reported [1]. Hypocalcemia has been found to be significantly associated with PD-1 inhibitor pembrolizumab treatment in a recent meta-analysis by Manohar et al. [5], notably as rare (11 out of 604 patients) grade 1–3 adverse events, but the pathomechanism is usually unknown. To our knowledge, there are only two case reports to date on severe hypocalcemia with confirmed hypoparathyroidism - one in a melanoma patient associated with nivolumab/ipilimumab treatment [6] and another in a small cell lung cancer patient on nivolumab treatment [7].

Normally, the calcium sensing receptor (CaSR) of parathyroid glands stimulate the release of PTH by the parathyroid glands upon sensing of low blood calcium. PTH in turn increases renal tubular Ca^2+^ reabsorption, enhances 1,25(OH)_2_D_3_ synthesis and increases Ca^2+^ release from bone, thus restoring normal calcium levels. CaSR autoantibodies and CaSR-activating autoantibodies have previously been identified in patients with idiopathic hypoparathyroidism and autoimmune polyendocrine syndrome type 1 (APS1) [8] and have also been described in a patient treated with nivolumab [7]. Various pathomechanisms of irAEs have been described and proposed [4, 9]. One prominent mechanism is the loss of peripheral tolerance to autoantigens where, for example, T cell clones react against antigens in tumors that are also present in healthy tissue [10, 11]. This putative mechanism includes cross-presentation of tumor-antigens that leads to expansion of T cell clones, which then infiltrate healthy tissue and cause inflammation [9]. Also, autoantibodies have been found in irAEs induced by ICI therapy including in a patient with cerebral vasculitis [12], or autoimmune bullous skin disorders [13]. Autoimmune hypoparathyroidism can be caused by either immune-mediated destruction or by hyperactivation of the CaSR by activating autoantibodies. Interestingly, we find that the hypoparathyroidism in the described patient was dependent on immune-suppressive medication and therefore was rather inflammation-mediated. Since we could not detect significant titers of anti-CaSR antibodies in our patient, it seems the pathomechanism is probably distinct from the one seen in sporadic hypoparathyroidism.

## Conclusion

This case describes a rare immune-related endocrinopathy in a patient treated with combination immune checkpoint blockade for melanoma. Therefore, physicians caring for cancer patients treated with ICI should be aware of such irAEs. We also demonstrate that this patient has an inflammation -driven and not an autoantibody-mediated irAE. Our case report therefore also provides a new insight into the pathomechanism of this rare side effect of ICI treatment.

## Abbreviations

CaSR: Calcium sensing receptor
CT: Computed tomography
CTLA-4: Cytotoxic T-lymphocyte-associated protein-4
irAE: immune-related Adverse Event
mAb: monoclonal antibody
PD-1: Programmed Death-1
PD-L1: Programmed Death Ligand-1
TNFα: Tumor Necrosis Factor α
